# The independent prokaryotic origins of eukaryotic fructose-1, 6-bisphosphatase and sedoheptulose-1, 7-bisphosphatase and the implications of their origins for the evolution of eukaryotic Calvin cycle

**DOI:** 10.1186/1471-2148-12-208

**Published:** 2012-10-22

**Authors:** Yong-Hai Jiang, De-Yong Wang, Jian-Fan Wen

**Affiliations:** 1 State Key Laboratory of Genetic Resources and Evolution, Kunming Institute of Zoology, Chinese Academy of Sciences, Kunming, Yunan 650223, China; 2 Graduate School of the Chinese Academy of Sciences, Beijing 100039, China

**Keywords:** FBPase, SBPase, F/SBPase, Evolution, Calvin cycle

## Abstract

**Background:**

In the Calvin cycle of eubacteria, the dephosphorylations of both fructose-1, 6-bisphosphate (FBP) and sedoheptulose-1, 7-bisphosphate (SBP) are catalyzed by the same bifunctional enzyme: fructose-1, 6-bisphosphatase/sedoheptulose-1, 7-bisphosphatase (F/SBPase), while in that of eukaryotic chloroplasts by two distinct enzymes: chloroplastic fructose-1, 6-bisphosphatase (FBPase) and sedoheptulose-1, 7-bisphosphatase (SBPase), respectively. It was proposed that these two eukaryotic enzymes arose from the divergence of a common ancestral eubacterial bifunctional F/SBPase of mitochondrial origin. However, no specific affinity between SBPase and eubacterial FBPase or F/SBPase can be observed in the previous phylogenetic analyses, and it is hard to explain why SBPase and/or F/SBPase are/is absent from most extant nonphotosynthetic eukaryotes according to this scenario.

**Results:**

Domain analysis indicated that eubacterial F/SBPase of two different resources contain distinct domains: proteobacterial F/SBPases contain typical FBPase domain, while cyanobacterial F/SBPases possess FBPase_glpX domain. Therefore, like prokaryotic FBPase, eubacterial F/SBPase can also be divided into two evolutionarily distant classes (Class I and II). Phylogenetic analysis based on a much larger taxonomic sampling than previous work revealed that all eukaryotic SBPase cluster together and form a close sister group to the clade of epsilon-proteobacterial Class I FBPase which are gluconeogenesis-specific enzymes, while all eukaryotic chloroplast FBPase group together with eukaryotic cytosolic FBPase and form another distinct clade which then groups with the Class I FBPase of diverse eubacteria. Motif analysis of these enzymes also supports these phylogenetic correlations.

**Conclusions:**

There are two evolutionarily distant classes of eubacterial bifunctional F/SBPase. Eukaryotic FBPase and SBPase do not diverge from either of them but have two independent origins: SBPase share a common ancestor with the gluconeogenesis-specific Class I FBPase of epsilon-proteobacteria (or probably originated from that of the ancestor of epsilon-proteobacteria), while FBPase arise from Class I FBPase of an unknown kind of eubacteria. During the evolution of SBPase from eubacterial Class I FBPase, the SBP-dephosphorylation activity was acquired through the transition “from specialist to generalist”. The evolutionary substitution of the endosymbiotic-origin cyanobacterial bifunctional F/SBPase by the two light-regulated substrate-specific enzymes made the regulation of the Calvin cycle more delicate, which contributed to the evolution of eukaryotic photosynthesis and even the entire photosynthetic eukaryotes.

## Background

**Figure 1:**
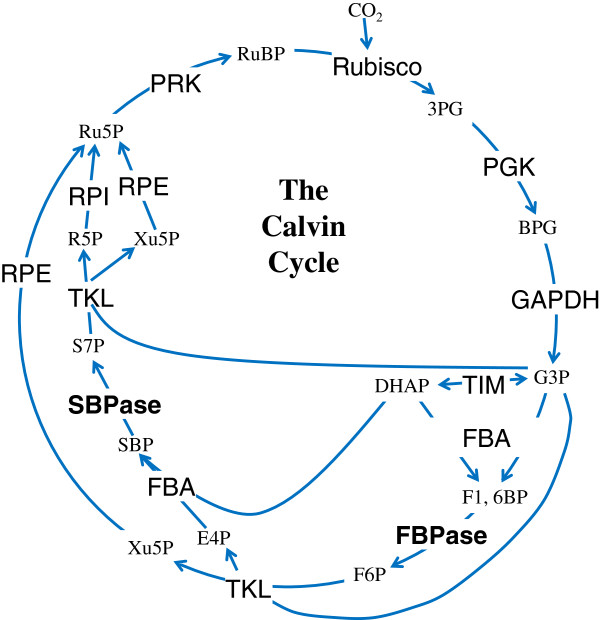
The schematic representation of eukaryotic Calvin Cycle.

The Calvin cycle is one of the five carbon fixation pathways in prokaryotes and the only one in photosynthetic eukaryotes [1]. It consists of 13 biochemical reactions catalyzed by 11 enzymes in the chloroplast of plants (Figure 1). In photosynthetic eubacteria, however, only 10 enzymes take part in this pathway because a dual-enzyme called fructose-1, 6-bisphosphatase/sedoheptulose-1, 7-bisphosphatase (F/SBPase) has the bifunction of both fructose-1, 6-bisphosphatase (FBPase) and sedoheptulose-1, 7-bisphosphatase (SBPase) [2,3]. Thus, SBPase, which catalyzes the dephosphorylation of sedoheptulose-1, 7-bisphosphate (SBP) into sedoheptulose-7-phosphate (S7P) and inorganic phosphate (Pi), is specific to the eukaryotic Calvin cycle. SBPase was also suggested to play vital roles in regulating the Calvin cycle pathway [4,5], improving photosynthetic capacity [6], and adapting to tolerate high temperature [7,8]. In spite of these important functions, this enzyme is one of the three Calvin cycle enzymes whose evolutionary origins are still unclear (the other two are fructose-1, 6-bisphosphate aldolase (FBA) and ribose 5-phosphate isomerase) [9]. Based on the sequence similarity between the two enzymes, Raines et al. [10] once speculated that the chloroplast FBPase and SBPase either arose from a common bisphosphatase progenitor, or diverged from a bifunctional F/SBPase, or evolved convergently. Later, the second scenario was supported by some phylogenetic analyses, which suggested that FBPase and SBPase were likely derived from a common eubacterial or mitochondrial bifunctional F/SBPase and subsequently acquired their respective substrate specificities [9,11]. However, actually no specific affinity between SBPase and eubacterial FBPase or F/SBPase can be seen on these previous phylogenetic trees. Moreover, according to this scenario it is hard to explain the fact that SBPase and/or F/SBPase are/is absent from most extant nonphotosynthetic eukaryotes [12]. Therefore, despite the critical importance of SBPase and FBPase in eukaryotic photosynthesis, their origins and evolutionary relationship remain uncertain so far. However, the knowledge about these is of singnicance in understanding the evolution of the Calvin cycle and photosynthesis. In the present work, we resolved to perform a comprehensive phylogenetic analysis and motif analysis using a much larger dataset (especially including much more diverse eubacterial lineages than previous work) to explore this issue. Our results showed that eukaryotic chloroplastic FBPase and SBPase have two distinct bacterial origins. In addition, the implications of the origins of the two specific light-regulated enzymes for the evolution of the eukaryotic Calvin cycle are also discussed.

## Results

### Eubacterial F/SBPase can be classified into two classes

Prokaryotic Class I and Class II FBPase are distantly related to each other [13]. Class I contains typical FBPase domain [14], while Class II contains FBPase_glpX domain [15]. When we searched against Pfam database to identify the two kinds of domains in all the available eubacterial F/SBPase whose dual function has been experimentally determined, eight sequences, six of which are from proteobacteria and two from cyanobacteria, were obtained. For all the eight sequences, one significant Pfam-A match but no Pfam-B match for each sequence was found. For the two sequences from cyanobacteria, one insignificant Pfam-A match for each was also found, but the E-values are very high (0.26 and 0.2, respectively), and the alignment are too short (about 40 amino acid in length), so the insignificant Pfam-A match was excluded from further analysis. The final results of our Pfam analysis showed that only the six proteobacterial F/SBPase each contains a typical FBPase domain, while the two cyanobacterial F/SBPase each contains a FBPase_glpX domain (Table 1). Because of the two different kinds of domains they contain, we propose that, according to the classification standard of prokaryotic FBPase, eubacterial F/SBPase can also be divided into Class I F/SBPase (having FBPase domain) and Class II F/SBPase (having FBPase_glpX domain).

**Table 1:** F/SBPases from proteobacteria and cyanobacteria contain distinct domains

**Organism**		**Accession**	**Domain**	**References**
proteobacteria	*Ralstonia eutropha*	CAJ96179	FBPase	[3]
	*Rhodobacter sphaeroides*	YP_001170173 YP_001168907	FBPase	[16]
	*Xanthobacter flavus*	P23014	FBPase	[17]
	*Rhodobacter capsulatus*	AAC32305	FBPase	[18]
	*Nitrobacter vulgaris*	AAA25505	FBPase	[19]
cynaobacteria	*Synechocystis sp* PCC 6803	BAA17988	FBPase_glpX	[20]
	*Synechococcus* PCC 7942	BAA11934	FBPase_glpX	[21]

### Phylogenetic correlation among eukaryotic SBPase, FBPase, and eubacterial class I FBPase, F/SBPase

**Figure 2:**
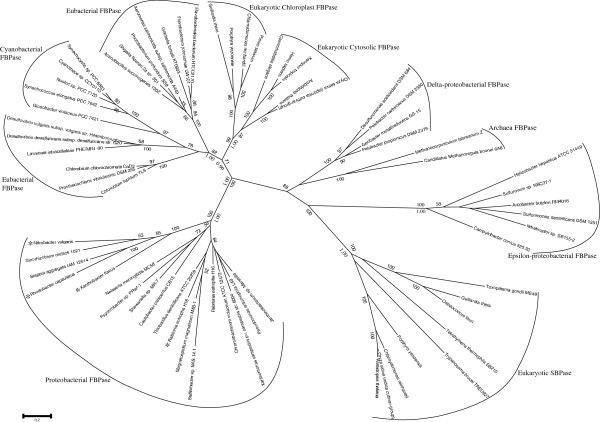
**The phylogenetic tree of FBPase, F/SBPase and SBPase based on 319 amino acid positions.** The sequences used include 53 eubacterial and eukaryotic Class I FBPase, 4 Class I F/SBPase, and 9 eukaryotic SBPase, and 2 archaebacterial FBPase were taken as outgroup. The consensus tree was constructed by the maximum likelihood method with 100 bootstrap replicates under RtREV model using PHYML. Only support values >50% for ML analysis and posterior probabilities from Bayesian analysis (below) of the big branches are shown. “*” indicates class I F/SBPase.

Our dataset for phylogenetic analyses comprised sequence data of eukaryotic SBPase, chloroplast and cytosolic FBPase, Class I FBPase from diverse bacteria (including green sulfur bacteria, CFB group bacteria, cyanobacteria, and five subgroups of proteobacteria), and Class I (proteobacterial) bifunctional F/SBPase we defined above (Class II FBPase and F/SBPase were excluded due to their distant relationship with these enzymes). Two archaeal homologs of Class I FBPase were used as outgroup. The sequence identities among these enzyme groups were about 30% (Table 2), according to the local blast results. ML and Bayesian analyses produced similar trees with minor differences in tree topologies, and thus the ML tree is taken as the backbone tree which is displayed in Figure 2. On the tree, although the support values are generally not high, which is probably mainly due to the far evolutionary distances among these sampled organisms, it can be found that: 1) all Class I F/SBPase we defined above group with the Class I FBPase of alpha-, beta- and some gamma-proteobacteria; 2) all eukaryotic SBPase cluster together and form a close sister group to the clade of epsilon-proteobacterial Class I FBPase; while 3) all eukaryotic chloroplast FBPase group together with cytosolic FBPase, forming another distinct clade, which then groups with the Class I FBPase of diverse eubacteria. Moreover, our AU tests rejected the two alternative topologies containing a jointed clade of eukaryotic SBPase and FBPase to be sister group and further to cluster together either to epsilon-proteobacterial Class I FBPase or diverse eubacterial Class I FBPase at a significant level. Therefore, these results suggest that eukaryotic SBPase and FBPase did not diverge from a common eubacterial F/SBPase ancestor, but evolved from a kind of Class I FBPase similar to that of epsilon-proteobacteria and Class I FBPase of another unknown eubacterial lineage, respectively.

**Table 2:** The sequence identities among different enzyme groups

		**Sequence identity (%)**	
		**Low**	**High**
F/SBPase	SBPase	25.61	32.43
F/SBPase	FBPase	22.99	63.64
FBPase	SBPase	26.13	38.26

### Comparison of sequence motif distribution patterns among eukaryotic SBPase, FBPase, eubacterial class I FBPase and F/SBPase

**Figure 3:**
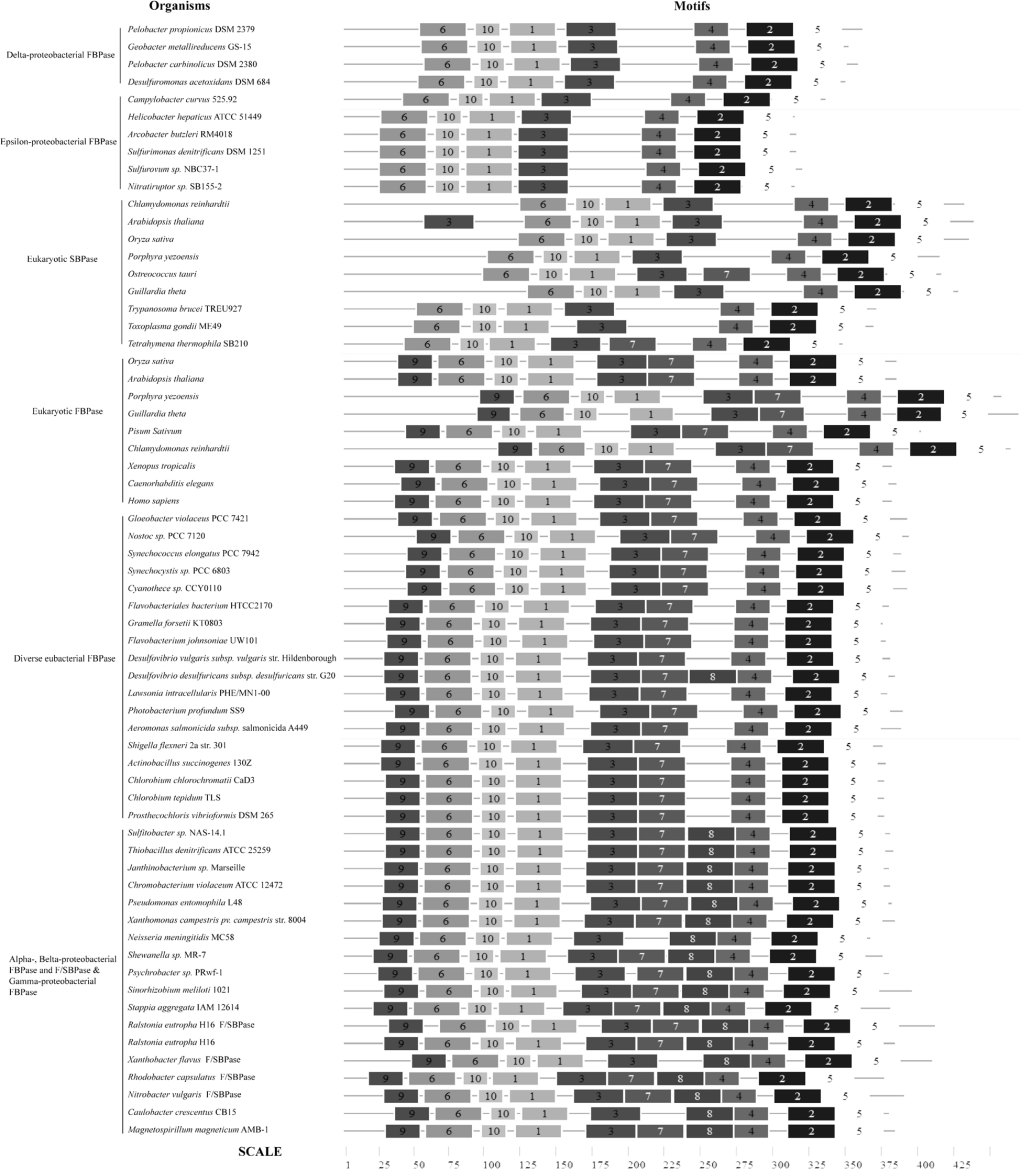
**Comparison of conserved motifs among eukaryotic FBPase, SBPase, eubacterial Class I FBPase, and F/SBPase.** The motifs were identified by MEME search tool. Each motif is represented by a numbered box. The length of box corresponds to the length of motif.

The MEME motif identification software was used to search the conserved motifs in eukaryotic SBPase, FBPase, and eubacterial Class I FBPase, F/SBPase. Totally, ten motifs, which were numbered from 1 to 10 according to their appearing order (Figure 3), were identified in these enzyme sequences. Among them, motif 1–6 and 10 are present in all the enzymes, while motif 7–9 have a complex distribution pattern: they are absent simultaneously from both eukaryotic SBPase and epsilon-proteobacterial Class I FBPase (only with the exception that the SBPase of *Ostreococcus tauri* and *Tetrahymena thermophila* SB210 have motif 7); two of them, motif 7 and 9, are present simultaneously both in eukaryotic FBPase and FBPase of diverse eubacteria except epsilon- and delta- proteobacteria, and the other one, motif 8, however, is almost restricted to alpha-, belta- and gamma-proteobacterial Class I FBPase and F/SBPase (Figure 3). These results suggest that in terms of motif distribution, eukaryotic SBPase is most similar to epsilon-proteobacterial Class I FBPase, while eukaryotic FBPase is closely related to the FBPase of diverse eubacteria except alpha-, belta- and gamma-proteobacteria. Therefore, the results of our motif analysis also support the phylogenetic correlations among these enzymes revealed above.

## Discussion

### Two evolutionarily distant classes of eubacterial F/SBPase

Until now, bifuntional F/SBPase have only been found in two bacterial lineages -- proteobacteria and cyanobacteria. Our domain analysis showed that they possess distinct domains corresponding to their source lineages, that is, proteobacterial F/SBPase contain the typical FBPase domain of Class I FBPase, while cyanobacterial F/SBPase have the FBPase_glpX domain of Class II FBPase. Therefore, for the first time, our work reveals that F/SBPase can also be divided into two classes, Class I F/SBPase with typical FBPase domain and Class II F/SBPase with FBPase_glpX domain, consistent with the classification scheme of FBPase. This classification is also supported by the following two facts: 1) our phylogenetic analysis showed that all proteobacterial F/SBPase (Class I) group with alpha-, beta- and gamma-proteobacterial Class I FBPase (Figure 2); and 2) cyanobacterial F/SBPase (Class II) are related to Class II FBPase according to the COG (Clusters of Orthologous Groups) database [22]. The Class I and Class II FBPase in eubacteria both use FBP as their specific substrate and participate in gluconeogenesis, while the two classes of bifunctional F/SBPase both use both FBP and SBP as their substrates and are both involved in the Calvin cycle [16,23]. Thus, we believe that it is reasonable to define these two classes of eubacterial F/SBPase to reflect the distinct features between them, and between them and eubacterial FBPase.

In spite of being involved in the same reaction and having the same layered αβαβα-type structure, the Class I and Class II FBPase have little sequence similarity [15]. Besides, the typical FBPase domain as well as the extended Li^+^-sensitive phosphate motif in Class I FBPase are absent from Class II FBPase [13,24]. Thus we can speculate that, even if they share a common ancestor, this ancestor might not be the last common ancestor, and the two classes of FBPase must have diverged a very long time ago and, therefore, are very distantly related. The same scenario can also be applied to explain the difference between the two classes of eubacterial F/SBPase defined in this work.

### The independent prokaryotic origins of eukaryotic FBPase and SBPase

Previous studies proposed that eukaryotic FBPase and SBPase possibly diverged from a common mitochondrial bifunctional F/SBPase ancestor [9,11]. However, although the support values of our tree are generally not high, which is usually a common phenomenon in the phylogenetic analyses of sampled organisms of far evolutionary distances, our phylogenetic analyses and motif analyses clearly showed that the two enzymes originated from two different eubacterial FBPase rather than have a common bifunctional ancestor, that is, eukaryotic SBPase share a common ancestor with the Class I FBPase of epsilon-proteobacteria (or probably originated from that of the ancestor of epsilon-proteobacteria), while eukaryotic FBPase originated from the Class I FBPase of an unknown kind of eubacteria.

Previous work did not show that eukaryotic SBPase are closely related to any particular eubacterial FBPase, and this was explained by the high degree of divergence between FBPase and SBPase [11]. However, the real reason is more probably the limited bacterial sequence sample included in their analyses. Because in our current analyses, once a significantly larger sequence samples from diverse groups of eubacteria were included, eukaryotic SBPase was showed to have a close affinity to epsilon-proteobacterial Class I FBPase. As for epsilon-proteobacterial Class I FBPase, at least the following two lines of evidence suggest it may be involved in other biologicl process rather than in the Calvin cycle. Firstly, ribulose-1, 5-bisphosphate carboxylase, the only CO_2_-fixing enzyme in the Calvin cycle, was not found in epsilon-proteobacteria [25], suggesting the Calvin cycle does not exist in this kind of bacteria at all; secondly, autotrophic epsilon-proteobacteria use the reductive tricarboxylic acid cycle for carbon assimilation [26,27], in which FBPase is not need. Thus, although there is no functionally experimental identification and characterization of the enzyme epsilon-proteobacterial Class I FBPase yet, this enzyme is unlikely to catalyze the dephosphorylation of SBP as efficiently as F/SBPase does in the Calvin cycle of other eubacteria. Therefore, it is more likely that epsilon-protoebacterial Class I FBPase is a gluconeogenesis-specific enzyme. Thus, we speculate that eukaryotic SBPase might evolve from a kind of gluconeogenesis-specific Class I FBPase similar to that of epsilon-proteobacteria through an unclear mechanism (probably lateral gene transfer).

### The mechanism of acquisition of the SBP-dephosphorylation activity of eukaryotic SBPase and the implications of the emergence of the two specific light-regulated enzymes (SBPase and FBPase) for the evolution of eukaryotic Calvin cycle

In extant eubacteria, no specific Class I or Class II FBPase uses SBP as significant substrate to catalyze its dephosphorylation, but F/SBPase can perform this task [11,15]. The SBP-dephosphorylation activity of F/SBPase should have arisen acommpanying the emergence of the Calvin cycle, and this must have occurred after the origin of gluconeogenesis because gluconeogenesis emerged prior to the Calvin cycle [28,29]. In addition, it was showed that the specificity of SBPase towards SBP versus FBP is high, but not absolute [30,31]. This means that not only eubacterial F/SBPase but also eukaryotic SBPase are “generalists”. It was demonstrated that a specific enzyme can convert into another one via “generalist” [32,33]. The evolution of beta-galactosidase from beta-glucuronidase is an example of such kind of conversion [34]. We consider that similar evolutionary mechanism might have occoured on eubacterial F/SBPase and eukaryotic SBPase. That is, during the origin of the Calvin cycle, the generation of the new intermediate SBP required a new enzyme activity to catalyze its dephosphorylation. Because of the similar structure of FBP and SBP, FBPase in some eubacteria might have a latent, promiscuous SBP-dephosphorylation activity, and thus the active site of ancestral promiscuous FBPase might gradually be modified to fit the new substrate SBP, and as a result the FBPase eventually evolved into F/SBPase in eubacteria and into SBPase in photosynthetic eukaryotes. For photosynthetic eukaryotes, the ancestral promiscuous FBPase might be acquired from the ancestor of extant epsilon-proteobacteria.

A cyanobacterial core of the plant Calvin cycle enzymes has been proved by previous phylogenetic analyses [35]. It has also been suggested that most Calvin cycle enzymes in photosynthetic eukaryotes are derived either from endosymbiotic gene transfer or from cytosolic isoform gene duplication [9]. However, our present work show that SBPase share a common ancestor with the Class I FBPase of extant epsilon-proteobacteria instead of arising through functional specialization of the eubacterial bifunctional F/SBPase. The Calvin cycle is regulated by light through two ways: 1) reversible dissociation of glyceraldehyde-3-phosphate dehydrogenase/CP12/phosphoribulokonase complex mediated by NADP(H) [36], which is conserved from cyanobacteria to photosynthetic eukaryotes [37]; 2) reductive activation of key enzymes (FBPase, SBPase, phosphoribulokonase, glyceraldehyde-3-phosphate dehydrogenase and ribulose bisphosphate carboxylase/oxygenase activase) by ferredoxin/thioredoxin system [38], which is only found in photosynthetic eukaryotes. F/SBPase is not a light-regulated enzyme in cyanobacteria [39], while both chloroplast FBPase and SBPase are light-regulated enzymes in photosynthetic eukaryotes. These suggest that during the origin of eukaryotic chloroplast from endosymbiotic cyanobacteria, the appearance of the two light-regulated enzymes (chloroplast FBPase and SBPase) resulted in the emergence of another light-regulation mechanism, which made the regulation of the Calvin cycle more delicate in the evolution of photosynthesis, and further made contribution to the evolution of the entire photosynthetic eukaryotes.

## Conclusions

Our work, for the first time, indicated that eubacterial bifunctional F/SBPase can be classified into two evolutionarily distant classes: Class I F/SBPase with typical FBPase domain and Class II F/SBPase with FBPase_glpX domain. We found that eukaryotic FBPase and SBPase do not diverge from either of the two classes of F/SBPase but have two independent origins: SBPase share a common ancestor with the gluconeogenesis-specific Class I FBPase of extant epsilon-proteobacteria (or probably originated directly from that of the ancestor of extant epsilon-proteobacteria), while FBPase arise from Class I FBPase of an unknown kind of eubacteria. Our analysis further indicated in the evolution of SBPase from eubacterial Class I FBPase, by the mechanism of transition “from specialist to generalist” SBPase acquired its SBP-dephosphorylation activity. During the origin of chloroplasts from cyanobacteria, the evolutionary substitution of the endosymbiotic-origin cyanobacterial bifunctional F/SBPase by the two light-regulated substrate-specific enzymes (FBPase and SBPase) made the regulation of the Calvin cycle more delicate, which contributed to the evolution of eukaryotic photosynthesis and even the entire photosynthetic eukaryotes.

## Methods

*Escherichia coli* Class I FBPase protein sequence (GenBank: NP_757176) was used as query to blastp search against the entire NCBI microbial genome database to get prokaryotic Class I FBPase homologs. The Eubacterial F/SBPase sequences used in this work were collected from the published papers [3,17-19], and the representative eukaryotic FBPase and SBPase sequences used were arbitrarily extracted from GenBank based on their annotation information.

For the obtained eubacterial F/SBPase protein sequences, Pfam database (version 25.0) [40] was used to perform their domain analyses.

Conserved motifs among Class I FBPase, F/SBPase, and SBPase were identified by using the protein motif prediction program MEME version 4.1.0 [41] under maximum number of motifs - 10.

The sequence identities among different enzyme groups used for phylogenetic analysis were directly derived from the tabular format of the local blast results.

To carry out phylogenetic analysis, multiple sequence alignments were performed with CLUSTAL X (version 1.8.3) [42] under default settings followed by manual refinement (Additional file 1). The best fitting model RtREV was selected by MODELGENERATOR (version 0.84) [43]. Maximum-likelihood and Bayesian trees were reconstructed using selected model. The proportion of invariable sites and gamma parameter were estimated by PHYML version 2.4.5 [44] for ML tree searches, with 100 bootstrap replicates. Bayesian analysis was performed with MrBayes (version 3.0b4) [45] initiating from a random starting tree, and running four chains simultaneously for 4,000,000 generations with sampling trees every 100 generations. The first 10,000 trees were discarded as the burn-in, and the posterior probabilities were calculated from the remaining 30,000 trees.

Two alternative topologies constraining the SBPase and eukaryotic FBPase as sister branches were generated with TreeView (version 1.6.6) [46]. Tree-puzzle (version 5.2) [47] was used to calculated the likelihoods with WAG model and Consel [48] was selected to perform the approximately unbiased (AU) tests.

## Abbreviations

FBP: Fructose-1, 6-bisphosphate; SBP: Sedoheptulose-1, 7-bisphosphate; FBPase: Fructose-1, 6-bisphosphatase; SBPase: Sedoheptulose-1, 7-bisphosphatase; FBA: Fructose-1, 6-bisphosphate aldolase.

## Competing interests

The authors declare that they have no competing interests.

## Authors’ contributions

YHJ and DYW carried out the major analysis and drafted the manuscript, and JFW, supervised the work and revised the manuscript. All authors read and approved the final manuscript to be published.

## Supplementary Material

Additional file 1 Alignment of SBPase, class I FBPase and F/SBPase protein sequences which was used to construct the phylogenetic tree in phylip format. Click here for file
